# The Effects of Enriching Shortbread Cookies with Dried Sea Buckthorn Fruit on the Physicochemical and Sensory Properties

**DOI:** 10.3390/molecules29215148

**Published:** 2024-10-31

**Authors:** Mirosława Karpińska-Tymoszczyk, Magdalena Surma, Marzena Danowska-Oziewicz, Lidia Kurp, Monika Jabłońska, Karolina Kusek, Tomasz Sawicki

**Affiliations:** 1 Department of Human Nutrition, University of Warmia and Mazury in Olsztyn, Słoneczna 45f, 10-718 Olsztyn, Poland; madan@uwm.edu.pl (M.D.-O.); lidia.kurp@uwm.edu.pl (L.K.); monika.jablonska@uwm.edu.pl (M.J.); 2 Department of Plant Products Technology and Nutrition Hygiene, Faculty of Food Technology, University of Agriculture in Krakow, Balicka 122, 30-149 Krakow, Poland; magdalena.surma@urk.edu.pl (M.S.);

**Keywords:** cookies, sea buckthorn, polyphenols, antioxidant activity, acrylamide reduction, sensory quality, functional foods

## Abstract

The present study evaluated the physicochemical parameters, polyphenolics content, levels of heat-induced compounds (acrylamide, furfural, 5-hydroxymethylfurfural), antioxidant properties, as well as sensory quality of shortbread cookies enriched with dried sea buckthorn fruit (SBF). The SBF-enriched cookies were prepared by replacing 1, 2, 3, or 5% of the flour with dried sea buckthorn fruit. Our results showed the presence of 12 phenolic acids and 5 flavonoids in the SBF, while two phenolic acids and two flavonoids were detected in the cookies. Most of the compounds were identified in the cookies enriched with 5% SBF. Among the phenolic acids, benzoic acid was the most abundant, while among the flavonoids, quercetin was the most abundant. 5-hydroxymethylofurfural was not detected in any cookies, and the addition of SBF contributed to the presence of furfural in baked products and increased the amount of acrylamide, and their content increased with the amount of SBF addition. The addition of sea buckthorn fruit at 5% distinctly exceeded the benchmark level of acrylamide in the cookies and worsened their sensory quality. It should also be noted that SBF significantly (*p* < 0.05) improved the antioxidant potential determined by two tests, ABTS and DPPH. The SBF-enriched cookies were characterized by significantly higher values of crispness and browning index compared to the control cookies. The results of the study indicate that SBF can be successfully used as a component of bakery products. In conclusion, the cookies with SBF can show improved technological and functional properties and constitute an added value bakery product that could provide health benefits.

## 1. Introduction

Cookies are a highly popular snack food due to their sensory appeal and digestibility [1]. They represent a type of confectionery product, enjoyed by people in various social contexts [2]. Their popularity is attributed mainly to the simple production process and long shelf life. The basic ingredients used in the production of shortbread cookies are flour, fat, and sugar, which are perceived as nutritionally deficient by consumers. As a result, numerous research efforts in recent years have focused on enhancing the nutritional profile of these products [3]. In light of growing consumer awareness and increased knowledge about diet-related diseases, the demand for functional products is steadily increasing [2]. To improve the nutritional value of cookies, they can be enriched with additives containing bioactive components. One such addition is sea buckthorn berries (*Hippophae rhamnoides* L.).

Sea buckhorn is a berry-producing plant widely recognized for its medicinal and nutritional properties. The fruit are small, with a yellowish-to-orange hue, and contain a wide range of bioactive compounds [4]. Sea buckthorn berries are particularly rich in phenolic compounds, including flavonoids and phenolic acids. Key polyphenols identified in the fruit are gallic acids, catechin, quercetin, and kaempferol [5]. These compounds have been associated with numerous health benefits, i.e., antioxidant, anticancer, antihyperlipidemic, anti-obesity, anti-inflammatory, antimicrobial, and antiviral effects, making sea buckthorn a valuable functional food [6,7,8]. In addition to polyphenols, see buckthorn berries also contain other antioxidants such as vitamin C, vitamin E, and carotenoids. The European *Hippophae rhamnoides subspecies rhamnoides* contains approximately 360 mg of vitamin C /100 g of berries, while the Chinese *Hippophae rhamnoides subspecies sinensis* may reach up to 2500 mg/100 g [9]. This high vitamin C content is notable for its stability, even after cooking or drying [10]. The vitamin E content of sea buckthorn berries is 160 mg/100 g [9]. Sea buckthorn berries are a rich source of carotenoids such as zeaxanthin β-carotene, β-cryptoxanthin, lutein, lycopene and γ-carotene [11,12]. While sea buckthorn is also a source of other nutrients such as carbohydrates, amino acids and fatty acids [5,8,9], its polyphenolic profile is of particular interest due to its contributions to the plant’s health-promoting properties.

Bakery products may contain a number of compounds that are formed in them during heat treatment, such as acrylamide, hydroxymethylofurfural and their derivatives [13]. Food processing at elevated temperature has been reported as the main source of acrylamide for food products. Rearrangement of amino acids in heat-processed foods could also generate acrylamide [14]. However, the essential pathway of acrylamide formation is the Maillard reaction between reducing carbohydrates (glucose, fructose, etc.) and amino acids (especially asparagine) a reaction responsible for the formation of specific taste and colour [15,16]. Laboratory tests showed that acrylamide in the diet may lead to cancer in animals and potentially increase tumour risk for humans of all ages [17]. At the same time, the increasingly broader knowledge of consumers and existing trends for healthy, palatable, and natural functional foods force food producers to introduce modernized products enriched with plant and plant byproducts into the market. This might be beneficial from two points of view: increasing the functional properties of food products and decreasing some of the so-called food-borne toxins, such as acrylamide [17,18].

The objective of the presented study was to investigate the impact of the addition of dried sea buckhorn fruit (SBF) on the polyphenol profile, heat-induced compounds content, antioxidant properties, colour and sensory quality of shortbread cookies.

## 2. Results and Discussion

### 2.1. Texture of Shortbread Cookies

The results of the shortbread cookies hardness assessments are presented in Table 1. The hardness of the control biscuits was 21.07 N, while that of the biscuits with the lowest addition of dried sea buckthorn fruit (1%) was lower (18.90 N). However, this difference was not statistically significant. The higher addition of dried sea buckthorn fruit (2, 3, and 5%) resulted in a significant reduction in biscuit hardness, with the greatest effect observed with the 3% addition.

A similar relationship was noted by Janotková et al. [19] in cookies with the addition of SB biomass. According to those researchers, this phenomenon may be attributed to a gradual reduction in the gluten content, which limited the formation of gluten matrices. Furthermore, the limited activity of amylases in the dough, as well as the content of phenolic compounds, could also contribute to the decreasing hardness values [20]. Additionally, according to Assis et al. [21], texture parameters such as brittleness and hardness are desirable to be as low as possible. They play an important role in the evaluation of cookies, because of their close connection with consumer perception of freshness.

### 2.2. Colour Parameters

The colour of biscuits, and in fact their degree of browning, is related to their composition (low water content, high sugar content) and to reactions occurring during baking, i.e., Maillard reactions and sugar caramelisation [22]. The use of vegetable additives also significantly affects the colour of the finished baked goods [23].

Colour parameters of shortbread cookies changed statistically significantly depending on the amount of dried sea buckthorn fruit addition (Table 1). The colour parameters L* and b* significantly decreased in biscuits with sea buckthorn addition, and to the greatest extent in biscuits with the highest addition. The significant darkening of the colour of the biscuits is due to the colour of the additive used. The addition of sea buckthorn in the lower amounts (1–3%) resulted in the increase in the a* parameter at a similar level from 6.44 (control sample) to 7.83–8.43, while its 5% addition resulted in the distinctly greater change in this parameter to 10.59.

It was shown that the addition of sea buckthorn caused a significant reduction in the colour saturation (parameter C*) of the biscuits and to the greatest extent it was observed in the sample with 5% addition. The value of the parameter *h*—which indicates colour angle (colour shade)—in the control biscuits was 80.64, and the addition of sea buckthorn caused a significant reduction in the value of this parameter causing a colour shift towards red. The shortbread cookies with 1, 2, and 3% sea buckthorn addition showed changes in this parameter at a similar level, while they were significantly higher in samples with 5% addition.

The intensity of the brown colour of the biscuits, which is produced during their baking as a result of non-enzymatic browning reactions, can be determined by the browning index BI [23]. The shortbread cookies with a 1% addition of sea buckthorn showed a similar BI parameter as the control biscuits, while the higher additions resulted in a significantly higher degrees of browning.

Total difference in colour (ΔE) was the largest when the highest amount of sea buckthorn was added (5%). According to literature [24], if ΔE > 3, the difference in colour can be observed by the human eye, therefore all products (SB1, SB2, SB3, and SB5) showed visibly different colour than control cookies.

### 2.3. Total Phenolic Content

The total phenolic content (TPC) for both SBF and shortbread cookies with sea buckthorn added are presented in Table 2. The TPC in SBF was noted at the level of 13.00 mg GAE/g, which aligns with data presented by Criste et al. [25], who reported that TPC in aqueous extracts of four Romanian sea buckthorn cultivars ranged from 10.12 to 18.66 mg GAE/g. Similarly, Geng et al. [26] found comparable results in their analysis of Chinese sea buckthorn cultivars where vacuum freeze-drying produced dehydrated sea buckthorn with a soluble TPC of 15.0 mg GAE/g. The cited authors also pointed out that the level of bioactive compounds in sea buckthorn depends on the variety [25,26].

The TPC in the shortbread cookies enriched with SBF ranged from 0.57 to 0.96 mg GAE/g (Table 2). The lowest value of TPC was obtained for the control cookies, while the highest value was obtained for the cookies containing 5% SBF. The addition of SBF increased the phenolic content by 8.1%, 20.8%, 38.0%, and 40.6% for the SB1, SB2, SB3, and SB5 samples, respectively. To the best of our knowledge, there is no available data in the literature regarding TPC in bakery products fortified with SBF. However, Park and Joo [27] demonstrated that the addition of sea buckthorn leaf powder in the range of 3 to 9% in rye cookies increased TPC values. In conclusion, enrichment of shortbread cookies with SBF may noticeably increase their polyphenol content, thus improving the bioactive properties of the final products.

### 2.4. Profile of Polyphenols

The profile and content of polyphenols identified in SBF and shortbread cookies with the addition of this fruit are presented in Table 3. A total of 17 compounds were identified in the analysed samples, including 12 phenolic acids and 5 flavonoids. All of the detected compounds were present in the SBF, while only four polyphenols (vanillic acid, benzoic acid, rutin and quercetin) were identified in the shortbread cookies with the additions of the dried sea buckthorn fruit. No polyphenolic compounds were identified in the control sample, which was prepared without the addition of SBF. The predominant polyphenolic group identified in SBF samples was phenolic acids, which constituted 63.4% of the total polyphenolic compounds. The most abundant phenolic acid was gentistic acid, which contributed 14.6% of the total polyphenolic content. The flavonoid with the highest concentration in SBF was rutin, representing 19.3% of the total flavonoid content. Danielski and Shahidi [28] using HPLC-UV-MS-TOF method found four free phenolic compounds (*o*-methylgallic acid, ferulic acid, apigenin, and ellagic derivative IV) in sea buckthorn pomace and nine (*o*-methylgallic acid, ferulic acid, hydroxycaffeic acid, *p*-coumaroyl malonyldihexoside, apigenin, (+)-catechin, catechin-*O*-dihexoside, ellagic acid pentoside, and A-type procyanidin) in sea buckthorn seeds.

In the current study, the content of polyphenolic compounds was approximately 13 and 2 times higher than that observed in the sea buckthorn pomace and seeds [28], respectively. When considering the sum of the polyphenol content in all analysed fractions (free, esterified, and insoluble-bound) in the study by Danielski and Shahidi [28], the concentration of polyphenols in sea buckthorn products was approximately six times lower for pomace and one and a half times lower for seeds when compared to our findings. The observed differences in polyphenolic profiles and contents in sea buckthorn may be attributed, among others, to the specific part of the plant, the extraction method used, and the analytical techniques employed [29]. Additionally, the variation in the number of identified compounds may be related to the diversity of sea buckthorn varieties [25].

The addition of sea buckthorn fruits to the shortbread cookies resulted in an increase in the content of quercetin and rutin (Table 3). The polyphenolic profile of the cookies was predominantly characterized by quercetin, contributing between 57.4% and 77.8% of the total polyphenols content. The second flavonoid identified in the analysed cookies, rutin, accounted for an average of 23.2%. Notably, phenolic acids, specifically vanillic and benzoic acids, were only detected in the cookies enriched with 5% SBF. The contents of these phenolic acids were 2.77 and 19.37 μg/g, respectively, representing 2.3% and 15.7% of the sum of polyphenols.

Despite the increased polyphenol content observed in the shortbread cookies enriched with sea buckthorn, the range of identified compounds was surprisingly limited when compared to the polyphenol profile of dried SBF. This may be due to a number of factors. First, the high temperature during baking may result in the degradation of polyphenolic compounds, reducing their presence in the final product [30]. Previous studies have shown that baked products undergo a significant reduction in phenolic compound levels due to depolymerisation of polyphenols and decarboxylation of phenolic acids during heat treatment. In addition, the difference in polyphenol levels under heat can be influenced by various reactions, such as oxidative degradation of phenolic acids and release of free acids from their conjugated forms. It is also possible that polyphenols might become bound or dissolved in the cookie matrix, which could influence the extraction process and make them less detectable [31,32]. Finally, the relatively low concentration of SBF added into the cookies may also hinder the availability or stability of polyphenols, making their detection more challenging despite the use of sensitive HPLC-MS method [30,33].

### 2.5. Antioxidant Activity

To assess changes in the antioxidant activity of cookies enriched with sea buckthorn fruit, spectrophotometric assays (DPPH and ABTS) were used (Table 2). The dried sea buckthorn fruit demonstrated a strong antioxidant capacity, with DPPH activity of 78.19 µm TE/g and ABTS activity of 88.27 µm TE/g. These values confirm the high antioxidant potential of SBF and highlights its suitability as a food functional ingredient. Previous studies have also examined antioxidant activity of sea buckthorn, however direct comparisons are challenging. For instance, one study expressed DPPH results as mg ascorbic acid equivalent per kg FW [34], which is not directly comparable to our results. Dienaitė et al. [35], in turn, demonstrated significant antioxidant activity of defatted sea buckthorn berry pomace, but the fact that the authors focused on pomace rather than whole fruit limits the applicability of those findings to our work.

The control cookies showed no detectable DPPH activity and minimal ABTS activity (0.14 µm TE/g). Adding SBF to the cookie matrix significantly enhanced the antioxidant properties, with increases corresponding to the concentration of SBF. Cookies with 1% SBF displayed a slight rise in DPPH activity, while their ABTS activity remained similar to the control. With 2% SBF, both DPPH and ABTS activities increased to 0.85 µm TE/g and 1.24 µm TE/g, respectively, and this trend continued in the 3% SBF-enriched cookies. The highest antioxidant activity was observed in the cookies with 5% SBF, where DPPH reached 1.95 µm TE/g and ABTS 3.10 µm TE/g. This demonstrates a clear, dose-dependent enhancement of antioxidant capacity with SBF addition. The study on wheat-flour cookies with 5% sea buckthorn reported an ABTS value of 4.99 µmol/g dry matter [17]. Park and Joo [27] showed that shortbread cakes with sea buckthorn leaf powder exhibited significantly higher DDPH radical scavenging capacity than control cakes. Variations in cookie composition, the specific form of sea buckthorn used, and processing methods may contribute to differences in antioxidant activity of the cookies, but all studies consistently demonstrate that sea buckthorn significantly enhances the antioxidant properties of baked products.

Our study showed a strong positive correlation between TPC and ABTS (*r* = 0.870; *p* < 0.05) and between TPC and DDPH (*r* = 0.997; *p* < 0.05).

### 2.6. Heat-Induced Compounds of Shortbread Cookies

Table 4 shows the findings of our analysis of the concentration of acrylamide and furfural in shortbread cookies that had been enriched with dried sea buckthorn fruit. The lowest concentration of acrylamide was observed in the control sample, at 298 µg/kg. The highest concentration of acrylamide was observed in the cookies with 5% dried sea buckthorn fruit addition (SB5), reaching 729.60 µg/kg (Table 4). This result may be of interest to consider further. It seems that the addition of dried sea buckthorn fruit at each percentage level may have resulted in a statistically significant increase in its content relative to the control sample. The addition of 1% (SB1), 2% (SB2), and 3% (SB3) of dried sea buckthorn fruit resulted in 20%, 16%, and 19% increases in acrylamide content, respectively. On the other hand, the addition of 5% led to a notable increase in acrylamide content, reaching 145% in the control sample. The results obtained for the 5% additive are the only ones that are statistically significantly different from the other individual additives. The results obtained for additives 1%, 2%, and 3% are not statistically significantly different from one another.

In 2015, the European Food Safety Authority (EFSA) responded to a request from the European Commission by issuing a scientific opinion on the presence of acrylamide in food. To date, the data derived from human studies, including both epidemiological and biomarker-based investigations, remain insufficient for the assessment of dose-response relationships. Consequently, a dose–response relationship has been established based on the findings from animal studies [36]. Two distinct BMDL10 values (lower limit on the benchmark dose for a 10% response) have been proposed for acrylamide: 0.17 mg kg⁻¹ BW day⁻¹ for neoplastic effects in mice and 0.43 mg kg⁻¹ BW day⁻¹ for peripheral neuropathy in rats. The margin of exposure (MOE) for the cancer-related effects of acrylamide, defined as the ratio between the BMDL10 and the dietary exposure of the population, ranges from 425 for average adult consumers down to 50 for high consumers of toddlers. These ranges indicate a potential concern for public health [37].

Despite the absence of legal restrictions on acrylamide in food, the European Commission has approved acrylamide as a carcinogenic agent and established benchmark levels for the presence of acrylamide in foodstuffs, as set forth in Commission Regulation (EU) 2017/2158 [38]. Foodstuffs were classified into one of ten categories, one of which is cookies and wafers. It is recommended that the presence of acrylamide in this category should not exceed 350 µg/kg. It would be beneficial to ascertain the content of acrylamide in the samples, with or without dried sea buckthorn fruit, was found to be below or equal to the benchmark level in 1%, 2%, and 3% of the samples. In the case of a 5% addition of dried sea buckthorn fruit, the benchmark level was exceeded by more than twofold. It may be helpful to consider that the addition of a higher percentage of dried sea buckthorn fruit to cookies may result in an increased exposure to acrylamide.

Acrylamide (2-propeneamide) is a neurotoxic compound classified as a genotoxicant and as a “potential human carcinogen” by the International Agency for Research on Cancer (IARC) [39]. As a result of this classification led to the maximum workplace concentration list defining it as a Category III A2 substance [40]. Historically, contamination by acrylamide was only thought to be an issue in water, and its potential exposure to humans was not considered a significant concern. However, its discovery in food has prompted global attention [41] following the reports of Sweden’s National Food Administration (NFA) and Stockholm University in April 2002 that unusually high levels of acrylamide are formed when starch-based food, such as potatoes, are baked or fried [42]. It has been suggested that the most probable route regarding the formation of acrylamide was reported to be the reaction of free asparagine and reducing sugars during the processing of foods at high temperatures, specifically the Maillard reaction [43]. The impact of sea buckthorn addition on acrylamide formation in shortbread pastries may be significant due to its chemical properties, although research on this topic is scarce. It is essential to consider the potential effects of sea buckthorn on acrylamide formation, particularly in relation to its quantity. The first factor to consider is the antioxidant content of sea buckthorn. Sea buckthorn is a rich source of antioxidants, including vitamins C and E, carotenoids, flavonoids, and phenols. These antioxidants can reduce the formation of acrylamide, a toxic chemical formed during the baking process, by acting as free radical scavengers, which play a role in acrylamide formation processes. The higher the amount of sea buckthorn in the cakes, the higher the antioxidant content, which can lead to a reduction in acrylamide. The second factor to consider is the pH of the cake. Sea buckthorn has a naturally sour taste, indicating a low pH. Modifying the pH can impact the Maillard reaction, which is the primary mechanism of acrylamide formation. Reducing the pH can inhibit the formation of acrylamide because the Maillard reaction is more pronounced in an environment with a higher pH, i.e., alkaline. The higher sea buckthorn content, due to its higher acidity, may result in a lower pH of the dough, which should theoretically reduce the formation of acrylamide during baking. An additional factor to consider is the sugar content of sea buckthorn. Sea buckthorn contains sugars, including glucose and fructose, which are classified as reducing sugars. These sugars may participate in the Maillard reaction, which can result in the formation of acrylamide. An increase in the quantity of sea buckthorn may result in an elevated level of reducing sugars within the dough, which could potentially contribute to an augmented formation of acrylamide. This phenomenon may occur unless counterbalanced by the presence of other factors, such as antioxidants. Another factor that may be considered is the alteration of the baking parameters. The incorporation of an increased quantity of sea buckthorn fruit into a cake can also influence its moisture content and texture, which may subsequently affect the required baking time and temperature. Such alterations may indirectly influence the formation of acrylamide, as prolonged and intense baking is conducive to its production. The optimal amount of sea buckthorn may potentially minimize the formation of acrylamide. However, to ascertain this, further experimental studies would be required, taking into account all the aforementioned factors.

It would appear that thus far, only a limited number of studies have examined the use of sea buckthorn fruit in bakery products in relation to acrylamide levels. The authors demonstrated that the addition of 5% sea buckthorn fruit resulted in the formation of lower levels of acrylamide in the cookies, with a concentration of 136 µg/kg, in comparison to the control sample, which had an acrylamide content of 1291 µg/kg. This represented an 89% reduction [17]. In the other paper, the authors conclude that the amount of acrylamide detected in the cookie samples with sea buckthorn fruit addition reached exceedingly high values. In addition to the nutritional benefits, the incorporation of sea buckthorn pomace resulted in a significant increase in the formation of acrylamide, which was observed to be twice as high in wheat and triticale biscuits and up to four and a half times higher in rye biscuits. To reduce the formation of acrylamide, the asparaginase treatment of wet sea buckthorn pomace was employed, resulting in a significant reduction of acrylamide (30–60%) in the final biscuits [44].

The results of the current study indicate that the furfural content in the cookies ranged from not detected to 2.63 mg/kg (Table 4). It would appear that there is a correlation between the level of addition of dried sea buckthorn fruit to the dough formulation and the furfural content of the cookie samples, with the latter increasing in proportion to the former. The results for furfural showed some interesting statistically significant differences between samples. It would seem that the cookies enriched with 5% dried sea buckthorn fruit (2.63 mg/kg) (Table 4) exhibited the highest concentrations of furfural. The control cookies showed reductions in furfural levels of 56%, 41%, 25%, and 9%, respectively, in comparison to the SB1, SB2, SB3, and SB4 samples.

Furfural is a chemical compound that is formed during the heat treatment of carbohydrate-rich foods, particularly pentoses (five-carbon sugars) such as arabinose and xylose. Its formation is associated with the dehydration reaction of these sugars at elevated temperatures. The presence of furfural in food products can serve as an indicator of the extent of thermal reactions, but an excess of it is deleterious to health, as it can have negative effects at higher concentrations [45]. The impact of sea buckthorn incorporation on furfural concentration in shortbread cookies is contingent upon a number of variables. The sea buckthorn fruit contains sugars, including xylose and other pentoses, which are direct precursors of furfural. The incorporation of a greater quantity of sea buckthorn into the dough may result in an elevated level of pentoses, consequently leading to a more pronounced generation of furfural throughout the baking process. Accordingly, an increase in the quantity of sea buckthorn should theoretically result in elevated furfural levels. The incorporation of sea buckthorn can alter the structure and characteristics of the dough, which may subsequently influence the baking characteristics of the resulting cakes. The addition of sea buckthorn to the dough may necessitate an extended baking period or a higher temperature (due to the elevated moisture content), which could result in a more pronounced formation of furfural. This is the inaugural study to present the furfural level in shortbread cookies that have been enriched with dried sea buckthorn fruit. To date, no article has been identified that examines the use of sea buckthorn in bakery products in relation to furfural levels.

It would appear that there are currently no established guidelines or restrictions for furfural intake, in contrast to those that exist for HMF. It would be remiss of us not to mention that there have been instances where excessive consumption of this compound may result in a range of cause many adverse effects, including eye irritation, nausea, headaches and weakness [45]. The results of the analysis indicated that the HMF was not present in the cookies.

### 2.7. Sensory Analysis of Shortbread Cookies

Table 5 shows the results of sensory evaluation of shortbread cookies. It was observed that the cookies with SBF were characterised by a less even baking degree and a higher intensity of the browned dough aroma than the control cookies. The fatty aroma was the least perceptible in the cookies with the highest addition of sea buckthorn (SB5). The aroma of sea buckthorn fruit added at a level of 1% was not very intensely perceptible (score 3) and a higher addition resulted in a significant increase in perceptibility to a level of scores from 4.17 to 5.25. The highest score for crispness was given for the biscuits with 3% sea buckthorn (score 9.08) and it was similar to the control biscuits (score 8.92). The cookies with 1% and 2% added SB were scored the same for this attribute (both scores 8.17), while the highest amount of SB (5%) resulted in the biscuits being rated as significantly less crispy (score 7.75).

The addition of dried sea buckthorn fruit also influenced the intensity of the taste of the cookies, and this depended on the amount of their addition. The taste of sea buckthorn fruit in cookies with 1 and 2% addition was perceptible at a similar level, while in products with higher addition, it was significantly more perceptible. At the same time, the addition of the fruit reduced the intensity of the sweet taste in the cookies, and to the greatest extent it was noted in product with 5% of SB. In terms of overall quality, biscuits with 1–3% added SBF were evaluated similarly (scores 8.17–8.50) to control biscuits (score 9.08), while those with the highest SBF addition were scored significantly worse (score 6.17).

Our observations seem to confirm the results reported by other authors. A study by Sturza et al. [46] showed that the addition of dried sea buckthorn flour at a level of 2% improved the sensory quality, i.e., the appearance, colour, and texture of sponge cakes. According to the study by Janotková et al. [19] addition of buckthorn biomass as a flour substitute in different amounts (5%, 10%, 15%, and 20%) had a positive effect on the sensory quality of cereal biscuits.

## 3. Materials and Methods

### 3.1. Shortbread Cookies Preparation

Shortbread cookies were prepared according to the recipe given in Table 6. The following raw materials were used to prepare the biscuits: wheat flour type 450 (Gdańskie Młyny Sp. z o.o., Gdańsk, Poland), extra butter 82% fat (Mlekovita, Wysokie Mazowieckie, Poland), icing sugar (Diamant, Pfeifer and Langen Polska S.A., Poznań, Poland), eggs (Złota Nioska, Poultry firm Woźniak, Sp. z o.o., Rawicz, Poland), dried sea buckthorn fruit *(Hippophae rhamnoides*, Green Essence, Sp. z o.o., Pyrzyce, Poland).

The dried sea buckthorn berries were ground to powder form in a Robot Coupe R301 food processor, Ultra (France). All the ingredients were chopped on a pastry board to form sticky particles, then the dough was kneaded by hand, formed into a ball and placed in a cooling chamber at a temperature of 4 °C for 30 min. Then, the dough was rolled out to a thickness of 10 mm, and 60 mm diameter cookies were cut out with cutters and baked at a temperature of 200 °C for 7 min in a Rational SCCWE-101 steam convection oven (Landsberg am Lech, Germany). The cookies were then cooled to room temperature, and a random selection of them was packed into plastic food containers and left until used for texture, colour, and sensory evaluation. The remainder of the cookies were packaged in string bags and stored at the temperature of −20 °C until used for chemical analyses.

### 3.2. Texture Analysis

Cookies texture was measured using a TA.XT.plus universal texture analyser (Stable Micro System, Godalming, UK) equipped with a 50 kg load cell. A single cookie was cut with a Standard Blade Set (HDP/BS) at a speed of 0.5 mm/s and at a trigger force of 5 g. Measurements were taken on six cookies from each treatment. The cutting force was taken as an index of cookies’ hardness. The results were recorded with use of the Texture Expert version 1.22 software.

### 3.3. Colour Parameters

Colour of the cookies was analysed in the CIE Lab system (L*, a*, b*) using a Konica Minolta CR-400 chromameter (Osaka, Japan) with a measurement area of 8 mm. The chromameter was calibrated before measurements using a white standard plate with Y = 89.3, x = 0.3159, and y = 0.3225. The measurements were performed using illuminant D65, and standard observer 2°. Colour measurements were performed at three randomly selected locations on the surface of six cookies, and then the results were averaged. The parameters determined were L* (L* = 0 (black) and L* = 100 (white)), a* (+a* = redness and −a*= greenness), and b* (+*b* = yellowness and −b* = blueness). In addition, hue and chroma were calculated. Hue is a qualitative colour parameter that refers to an angular position around a point or axis on a colour space coordinate diagram. Chroma (saturation) is a quantitative colour parameter that can be defined as the strength of a hue.

The colour saturation C* of the cookies was calculated according to Equation (1)

(1) $$C^{*}=\sqrt{{a^{*}}^{2}+{b^{*}}^{2}}$$

The hue angle h* of the cookies was calculated according to Equation (2)

(2) $$h=\arctan\left(\frac{b^{*}}{a^{*}}\right)$$

The browning index BI was calculated according to Equations (3) and (4)

(3) $$BI=\frac{100(x-0.31)}{0.172}$$

where:

(4) $$x=\frac{a^{*}+{1.75L}^{*}}{{5.645L}^{*}+a^{*}-{3.012b}^{*}}$$

The ΔE difference between two colours was calculated according to Equation (5)

(5) $$\Delta E_{\mathrm{Lab}}=\sqrt{{{\left(\Delta L^{*}\right)+(\Delta a}^{*})}^{2}+(\Delta {b^{*})}^{2}}$$

### 3.4. Phenolic Extraction Procedure

The extraction of polyphenols from dried sea buckthorn fruit was conducted using a mixture of water and methanol in a ratio of 20:80 (*v/v*). Approximately 300 mg of the pulverised cookie sample was extracted by vortexing for 30 s with 1 mL of the aforementioned solvent. Subsequently, the mixture was subjected to sonication for 30 s, vortexed, sonicated, and subjected to centrifugation (Micro Star 30R, VWR, Radnor, PA, USA) for 10 min at 14,000 RPM and 4 °C. The resulting supernatant was collected in a 5 mL flask. This procedure was repeated five times with 1.0 mL residual solvent. The analysis was conducted in triplicate. The obtained extracts were stored at −24 °C until further analysis.

### 3.5. Total Phenolic Content (TPC) Analysis

Total phenolic content (TPC) was obtained using Folin’s phenol reagent according to the procedure described by Horszwald and Andlauer [47]. A mixture containing 15 μL of appropriately diluted extract fractions and 240 μL of Folin’s phenol reagent was placed into the wells of microplates and incubated for 10 min at room temperature (RT). Next, 15 μL of 20% sodium carbonate was added and shaken. Absorbance was measured at 765 nm using a microplate reader (FLUOstar Omega, BMG LABTECH, Ortenberg, Germany). The results were expressed as mg of gallic acid (GAE)/g of sample. The linearity range of the calibration curve was from 0.062 to 0.50 mg/mL (R^2^ = 0.999).

### 3.6. Determination of Phenols Profile by UHPLC-DAD-MS

The analysis of phenols was performed according to the methodology described by Sawicki et al. [48]. Phenols qualitative and quantitative determinations were carried out using a UHPLC system (Nexera XR, Shimadzu, Japan) coupled with a diode area detector (DAD) and mass spectrometer (LCMS-2020, Shimadzu, Japan). Measurement parameters were as follows: eluent 0.01% formic acid in water with 2 mM ammonium formate (A) and 0.01% formic acid in 95% acetonitrile solution with 2 mM ammonium formate (B); flow rate 0.15 mL/min; scanning in negative ionization; column Column C18 BEH (1.7 μm particle size; 100 × 2.1 mm; Waters, Warsaw, Poland); oven temperature 50 °C; sample injection volume 10 µL. An analysis was conducted in the selected ion monitoring mode (SIM). Analysed compounds were identified based on their qualitative ions, retention times and λmax value with the previously published data [49,50]. The quantity of phenols was calculated from the UHPLC-DAD-MS peak area against commercially available standards.

### 3.7. Antioxidant Activity

#### 3.7.1. ABTS Assay

The measurement was carried out according to the method described by Horszwald and Andlauer [47]. Briefly, ABTS+ solution was diluted with a water:methanol mixture (20:80, *v/v*) to an absorbance level of 0.70 ± 0.02 at 734 nm. For the spectrophotometric assay, 290 μL of the ABTS+ solution and 10 μL of the respective extract, Trolox or blank (80% methanol) were mixed, and absorbance was measured at 734 nm directly after 6 min incubation at 30 °C using a microplate reader (FLUOstar Omega, BMG LABTECH, Ortenberg, Germany). The response of the calibration curve was linear from 0.01 to 2.0 mM (R^2^ = 0.999). The results were expressed as μmol of Trolox equivalents (TE)/g of sample. Analysis was carried out in triplicate.

#### 3.7.2. DPPH Assay

Determination of antioxidant properties by the DPPH method was conducted according to the procedure developed by Horszwald and Andlauer [47]. The DPPH radical solution was prepared by dissolving 10 mg of DPPH in 250 Ml of 80% methanol. To perform the spectrophotometric test, 300 µL of DPPH solution and 20 µL of appropriately diluted sample or Trolox solution were mixed. The resulting mixture was left for 30 min at room temperature in the dark. Decreasing absorbance of the resulting solution was monitored at 517 nm using a microplate reader FLUOstar Omega (BMG LABTECH). The standard curve was plotted based on the length of the lag phase versus Trolox concentrations within the range of 0.01–2.0 Mm (R^2^ = 0.998). The results were expressed as μmol of TE/g of sample. Analysis was carried out in triplicate.

### 3.8. Determination of Acrylamide, Furfural, and HMF

#### 3.8.1. Acrylamide (QuEChERS Method)

A representative portion of the sample, comprising one gram, was placed into a 50-millilitre polypropylene (PP) centrifuge tube. Subsequently, 5 mL of distilled water, 5 mL of hexane, and 10 mL of acetonitrile were added to the mixture, which was then vigorously shaken for 1 min. Then, 1 g of sodium chloride and 4 g of magnesium sulfate were added, the tube was shaken for an additional minute, and centrifugation was performed (15 min, 10,733× *g*, 4 °C). In total, 6 millilitres of the supernatant was transferred into a PP 15-millilitre tube containing 0.15 g of primary secondary amine (PSA), 0.3 g of C18, and 0.9 g of magnesium sulphate. Following a 30-s period of agitation and 15-min centrifugation step (10,733× *g*, 4 °C), 4 mL of the extract was transferred from the upper layer and subjected to evaporation under an N_2_ stream until dryness. The resulting residues were dissolved in 100 μL of acetonitrile and subjected to analysis by HPLC-DAD (VWR HITACHI, LaChrom ELITE, Merck KGaA, Darmstadt, Germany) [51].

The analysis of acrylamide was performed according to the methodology previously described by Gumul et al. [51]. The chromatographic separation was conducted at room temperature using a mixture of 0.01 M sulfuric acid in water/methanol (97.5:2.5) in isocratic mode with a C18 reversed-phase column. A C18 column (Lichrospher^®^ 100, RP-18 end-capped, LiChroCART^®^ 4 mm ID × 250 mm, 10 µm, Merck KGaA, Darmstadt, Germany) was used, with a flow rate of 0.7 mL/min. Acrylamide was determined at a wavelength of 200 nm.

#### 3.8.2. Furfural and HMF

Ten millilitres of water were added to 2 g of cookie samples, and the solution was quantitatively transferred to a 25-mililitre volumetric flask. Subsequently, 0.25 mL of Carrez solution I and 0.25 mL of Carrez solution II were added. The volumetric flask was filled to the measuring mark with deionised water. In the case of infusions, 0.07 mL of Carrez solution I and 0.07 mL of Carrez solution II were added to 6.86 mL of the infusion solution. Reagent blank samples were prepared according to the appropriate methodology for all tested analytes [49].

The HMF and furfural analysis was conducted using a high-performance liquid chromatography-diode array detector (HPLC-DAD) system (VWR HITACHI, LaChrom ELITE, Merck KGaA, Darmstadt, Germany). The measurement parameters were as follows: eluent water/methanol (9:1, *v/v*), the flow rate 1 mL/min, UV detection at 285 nm, retention time 9.1 min, column RP-18 Lichrosphere (250 × 4 mm, 5 µm particle size) (Merck, Darmstadt, Germany) [51].

### 3.9. Sensory Analysis

The numerical interval scale ranging from 1 to 10 points was used to evaluate the sensory quality of the cookies [52]. The following characteristics were evaluated: evenness of baking (uneven degree of baking–even degree of baking); intensity of fatty, baked and sea buckthorn aroma (absent–very strong); crispness (hard–very crispy); intensity of fatty, baked, sweet, and sea buckthorn taste (absent–very strong); and overall quality (very poor–excellent).

The evaluation panel consisted of 12 persons (8 women and 4 men, aged 35–55), employees of the Faculty of Food Science of the University of Warmia and Mazury in Olsztyn (Poland). The panellists had 8–20 years of theoretical and practical experience with sensory procedures and evaluation of different food products with various methods and were trained in sensory analysis of food according to Standard ISO 8586: 2023 [53] and the method used to evaluate cookies. The sensory evaluation of the cookies was performed in the sensory analysis laboratory designed according to the ISO 8589: 2007 standard [54]. The samples were served to panellists at room temperature (20 ± 2 °C), on the white porcelain plates, in random order with a random three-digit code. Between the subsequent evaluations, the assessors were provided with water to neutralize the taste in the mouth. Each panellist carried out two tastings.

### 3.10. Statistical Analysis

The results were analysed statistically using Statistica version 13.3 software (TIBCO Software Inc., Tulsa, OK, USA). The mean values with standard deviations, significance of differences between means using the Tukey’s test and the Pearson’s correlations coefficients between the variables were determined. All tests were performed at significance level *p* < 0.05.

## 4. Conclusions

To the best of our knowledge, this is the first study representing the profile of polyphenol compounds in shortbread cookies enriched with the sea buckthorn. The addition of dried sea buckthorn fruit to cookies markedly elevated the polyphenol concentration in the final products and increased their antioxidant potential. The addition of dried sea buckthorn fruit significantly influenced the colour, texture and sensory quality of the cookies. The incorporation of SBF up to 3% was more favourable than 5% due to less colour change and better sensory quality. Cookies with 1–3% addition of dried sea buckthorn fruit were characterised by a significantly lower content of nutritionally unfavourable reaction products occurring during baking, such as acrylamide and furfural, than those with 5% addition. The presented findings can be considered as an optimization of shortbread cookies, which allowed to balance health benefits of cookies with formation of harmful compounds during baking.

## Figures and Tables

**Table 1:** The effect of the dried sea buckthorn fruit addition on hardness and colour parameters of shortbread cookies.

Samples	Hardness	Colour Parameters						
	[N]	*L* *	*a* *	*b* *	*C* *	*h*	BI	ΔΕ
C	21.07 ^c^ ± 1.60	68.48 ^d^ ± 2.94	6.44 ^a^ ± 1.76	38.99 ^d^ ± 0.64	39.56 ^d^ ± 0.62	80.64 ^c^ ± 2.55	87.55 ^a^ ± 7.15	-
SB1	18.90 ^bc^ ± 2.43	59.29 ^c^ ± 2.31	7.94 ^b^ ± 1.40	34.87 ^bc^ ± 1.04	35.79 ^bc^ ± 1.46	77.21 ^b^ ± 2.03	94.70 ^a^ ± 9.89	10.73 ^a^ ± 3.14
SB2	16.49 ^b^ ± 1.76	53.78 ^b^ ± 2.48	8.43 ^b^ ± 1.08	34.01 ^b^ ± 0.78	35.06 ^b^ ± 0.82	76.08 ^b^ ± 1.73	105.90 ^b^ ± 8.08	15.80 ^b^ ± 3.44
SB3	11.41 ^a^ ± 0.93	56.35 ^b^ ± 1.50	7.83 ^b^ ± 0.80	35.78 ^c^ ± 0.61	36.64 ^c^ ± 0.67	77.65 ^b^ ± 1.17	104.88 ^b^ ± 5.76	12.80 ^ab^ ± 3.15
SB5	17.71 ^b^ ± 0.83	40.24 ^a^ ± 3.05	10.59 ^c^ ± 0.62	25.99 ^a^ ± 2.03	28.09 ^a^ ± 1.76	67.71 ^a^ ± 2.41	116.36 ^c^ ± 5.86	31.36 ^c^ ± 3.77

The results are expressed as the mean ± SD; L*—lightness; a*—redness; b*—yellowness; C*—colour saturation; *h*—colour shade; BI—browning index; ΔΕ—total colour difference; ^a, b, c, d^—different letters in columns depict statistically significant differences (*p* < 0.05); C—control cookies (without addition of the dried sea buckthorn fruit); SB—shortbread cookies with the addition of the dried sea buckthorn fruit; The number (1, 2, 3, 5) denote the proportion of the dried sea buckthorn fruit incorporated into the cookies.

**Table 2:** The effect of the dried sea buckthorn fruit addition on the total phenolic content (TPC) and antioxidant activity of shortbread cookies.

Samples	Parameters		
	TPC	DDPH	ABTS
	(mg GAE/g)	(μm TE/g)	(μm TE/g)
Dried sea buckthorn fruit	13.00 ± 0.09	78.19 ± 4.86	88.27 ± 2.09
Type of cookies:			
Control (C)	0.57 ^a^ ± 0.04	nd	0.14 ^a^ ± 0.02
With 1% dried sea buckthorn fruit (SB1)	0.62 ^a^ ± 0.02	0.24 ^a^ ± 0.02	0.23 ^a^ ± 0.02
With 2% dried sea buckthorn fruit (SB2)	0.72 ^b^ ± 0.03	0.85 ^b^ ± 0.07	1.24 ^b^ ± 0.06
With 3% dried sea buckthorn fruit (B3)	0.92 ^c^ ± 0.05	1.87 ^c^ ± 0.04	1.48 ^c^ ± 0.07
With 5% dried sea buckthorn fruit (SB5)	0.96 ^c^ ± 0.02	1.95 ^c^ ± 0.12	3.10 ^d^ ± 0.02

The results are expressed as the mean ± SD; ^a, b, c, d^—different letters indicate statistically significant differences (*p* < 0.05) within the same column; TPC—total phenolic content; DDPH—DDPH radical scavenging assay; ABTS—ABTS radical scavenging assay; TE—Trolox equivalent; GAE—gallic acid equivalent; nd—not detected.

**Table 3:** The content and profile of polyphenols detected in the dried sea buckthorn fruit and shortbread cookies with the additions of the dried sea buckthorn fruit.

Phenolic Compounds	Samples					
	SBF	C	SB1	SB2	SB3	SB5
	Phenolic acids (μg/g of sample)					
gallic acid	105.57 ± 0.15	nd	nd	nd	nd	nd
gentisic acid	204.42 ± 1.97	nd	nd	nd	nd	nd
rosmarinic acid	105.70 ± 0.23	nd	nd	nd	nd	nd
salicylic acid	23.46 ± 0.07	nd	nd	nd	nd	nd
chlorogenic acid	5.74 ± 0.05	nd	nd	nd	nd	nd
vanillic acid	20.15 ^b^ ± 0.45	nd	nd	nd	nd	2.77 ^a^ ± 0.03
ellagic acid	85.61 ± 1.05	nd	nd	nd	nd	nd
*p*-coumaric acid	1.66 ± 0.07	nd	nd	nd	nd	nd
*m*-coumaric acid	98.51 ± 0.08	nd	nd	nd	nd	nd
*o*-coumaric acid	97.37 ± 0.13	nd	nd	nd	nd	nd
ferulic acid	63.76 ± 0.02	nd	nd	nd	nd	nd
benzoic acid	77.87 ^b^ ± 0.94	nd	nd	nd	nd	19.37 ^a^ ± 0.16
	Flavonoids (μg/g of sample)					
(+)-catechin	62.70 ± 0.68	nd	nd	nd	nd	nd
rutin	270.25 ^d^ ± 2.00	nd	20.45 ^a^ ± 0.03	22.15 ^a^ ± 0.05	23.73 ^ab^ ± 0.04	27.52 ^c^ ± 0.03
quercetin	109.06 ^d^ ± 0.12	nd	71.81 ^a^ ± 0.01	72.29 ^b^ ± 0.01	72.59 ^b^ ± 0.01	73.42 ^c^ ± 0.04
apigenin	7.95 ± 0.04	nd	nd	nd	nd	nd
naringenin	62.82 ± 0.00	nd	nd	nd	nd	nd
**Sum of polyphenols**	**1402.58 ^c^ ± 7.82**	**nd**	**92.26 ^a^ ± 0.04**	**94.43 ^a^ ± 0.05**	**96.29 ^a^ ± 0.05**	**123.07 ^b^ ± 0.26**

The results are expressed as the mean ± SD; ^a, b, c^—different letters in rows depict statistically significant differences (*p* < 0.05); values were expressed as micrograms per gram of sample (μg/g); nd—not detected; SBF—dried sea buckthorn fruit; C—control cookies (without additions of the dried sea buckthorn fruit); SB—shortbread cookies with the additions of the dried sea buckthorn fruit; The number (1, 2, 3, 5) denote the proportion of the dried sea buckthorn fruit incorporated into the cookies. The total amount of polyphenols are marked in bold.

**Table 4:** The content of reaction products occurring during heat treatment in dried sea buckthorn fruit and shortbread cookies with additions of dried sea buckthorn fruit.

Samples	Parameters	
	Acrylamide	Furfural
	[μg/kg]	[μg/kg]
SBF	754.70 ^c^ ± 14.75	2.90 ^e^ ± 0.08
Type of cookies:		
C	297.83 ^a^ ± 10.65	nd
SB1	356.50 ^b^ ± 1.60	1.28 ^a^ ± 0.04
SB2	347.30 ^b^ ± 17.20	1.72 ^b^ ± 0.25
SB3	353.90 ^b^ ± 7.10	2.17 ^c^ ± 0.03
SB5	729.60 ^c^ ± 21.10	2.63 ^d^ ± 0.14

The results are expressed as the mean ± SD; ^a, b, c, d, e^—different letters in columns depict statistically significant differences (*p* ≤ 0.05); nd—not detected; SBF—dried sea buckthorn fruit; C—control cookies (without addition of the dried sea buckthorn fruit); SB—shortbread cookies with the addition of the dried sea buckthorn fruit; The number (1, 2, 3, 5) denote the proportion of the dried sea buckthorn fruit incorporated into the cookies.

**Table 5:** The effect of the dried sea buckthorn fruit addition on the sensory quality of the cookies.

Attributes	Samples				
	C	SB1	SB2	SB3	SB5
Evenness of baking	8.08 ^c^ ± 0.79	6.08 ^ab^ ± 0.79	6.17 ^ab^ ± 0.94	6.67 ^b^ ± 0.94	5.17 ^a^ ± 1.27
Fatty aroma	5.92 ^b^ ± 0.90	5.33 ^ab^ ± 0.78	5.50 ^b^ ± 1.24	5.17 ^ab^ ± 1.27	4.17 ^a^ ± 0.94
Baked aroma	4.92 ^a^ ± 0.90	5.42 ^ab^ ± 0.51	6.58 ^c^ ± 0.90	6.25 ^bc^ ± 1.14	8.17 ^d^ ± 1.11
Sea buckthorn aroma	nd	3.00 ^a^ ± 0.74	4.17 ^b^ ± 1.27	5.25 ^b^ ± 0.62	4.42 ^b^ ± 1.31
Crispness	8.92 ^bc^ ± 0.51	8.17 ^ab^ ± 0.39	8.17 ^ab^ ± 0.72	9.08 ^c^ ± 0.90	7.75 ^a^ ± 0.76
Fatty taste	5.83 ^ab^ ± 0.58	5.02 ^ab^ ± 1.08	6.42 ^b^ ± 0.79	5.42 ^a^ ± 0.90	5.58 ^ab^ ± 0.79
Baked taste	4.25 ^a^ ± 0.62	4.42 ^a^ ± 1.44	6.67 ^b^ ± 1.56	6.33 ^b^ ± 1.23	8.17 ^c^ ± 1.19
Sweet taste	6.83 ^c^ ± 0.94	5.92 ^bc^ ± 1.56	5.08 ^b^ ± 0.90	5.00 ^b^ ± 0.74	3.67 ^a^ ± 0.98
Sea buckthorn taste	nd	3.50 ^a^ ± 0.67	4.58 ^a^ ± 1.24	6.33 ^b^ ± 1.23	7.50 ^b^ ± 1.31
Overall quality	9.08 ^c^ ± 0.51	8.33 ^bc^ ± 0.78	8.17 ^b^ ± 0.94	8.50 ^bc^ ± 0.90	6.17 ^a^ ± 0.72

The results are expressed as the mean ± SD; ^a, b, c, d^—different letters in columns depict statistically significant differences (*p* < 0.05); nd—not detected; C—control cookies (without additions of the dried sea buckthorn fruit); SB—shortbread cookies with the additions of the dried sea buckthorn fruit; The number (1, 2, 3, 5) denote the proportion of the dried sea buckthorn fruit incorporated into the cookies.

**Table 6:** The recipe formulation of control shortbread cookies and cookies enriched with dried sea buckthorn fruits.

Ingredient	The Level of White Flour Replacement with Dried Sea Buckthorn Fruits (%)				
	0	1	2	3	5
	Samples Codes				
	C	SB1	SB2	SB3	SB5
Wheat flour	49	48	47	46	44
Butter	30	30	30	30	30
Powdered sugar	16	16	16	16	16
Egg yolk	4.9	4.9	4.9	4.9	4.9
Dried sea buckthorn fruits (SBF)	0	1	2	3	5

## Data Availability

Data is contained within the article.

## References

[1] Najjar Z., Alkaabi M., Alketbi K., Stathopoulos C., Ranasinghe M. (2022). Physical Chemical and Textural Characteristics and Sensory Evaluation of Cookies Formulated with Date Seed Powder. Foods.

[2] Dziki D., Lisiecka K., Gawlik-Dziki U., Różyło R., Krajewska A., Cacak-Pietrzak G. (2022). Shortbread Cookies Enriched with Micronized Oat Husk: Physicochemical and Sensory Properties. Appl. Sci..

[3] Végh R., Csóka M., Stefanovits-Bányai É., Juhász R., Sipos L. (2023). Biscuits Enriched with Monofloar Bee Pollens: Nutritional Properties, Techno-Functional Parameters, Sensory Profile and Consumer Preference. Foods.

[4] Singh B., Oberoi S., Kaur A. (2024). Phenolic compounds in sea buckthorn (*Hippophaë rhamnoides* L.) amnd therir health-promoting activities: A review. Int. J. Food Sci. Technol..

[5] Jastrząb A., Skrzydlewska E. (2019). Composition and biomedical relevance of sea buckthorn. Acta Pol. Pharm. Drug Res..

[6] Piłat B., Zadernowski R. (2016). Sea buckthorn fruits (*Hippophaë rhamnoides* L.)—A rich source of biologically active compounds (in Polish: Owoce rokitnika (*Hippophhaë rhamnoides* L.)—Bogate źródło związków biologicznie aktywnych). Post. Fototer..

[7] Dubey R.K., Shukla S., Shukla V., Singh S. (2023). Sea Buckthorn: A Potential Dietary Supplement with Multifaceted Therapeutic Activities. Intell. Pharm..

[8] Tereshchuk L., Starovoytova K., Babich O., Dyshlyuk L., Sergeeva I., Pavsky V., Ivanova S., Prosekov A. (2020). Sea Buckthorn and Rosehip Oils with Chokeberry Extract to Prevent Hypercholesterolemia in Mice Caused by a High-Fat Diet in Vivo. Nutrients.

[9] Bal L.M., Meda V., Naik S.N., Satya S. (2011). Sea buckhorn berries: A potential source of valuable nutrients for nutraceuticals and cosmeceuticals. Food Res. Int..

[10] Urbaniak S., Kaźmierczak-Barańska J., Karwowski T. (2019). Sea buckthorn (*Hippophhaë rhamnoides* L.) as a treasure trove of vitamin C (in Polish: Rokitnik zwyczajny (*Hippophhaë rhamnoides* L.) jako skarbnica witaminy C). Post. Biochem..

[11] Mihalcea L., Turturică M., Ghinea I.O., Barbu V., Ioniţă E., Cotârleț M., Stănciuc N. (2017). Encapsulation of carotenoids from sea buckthorn extracted by CO2 supercritical fluids method within whey proteins isolates matrices. Innov. Food Sci. Emerg. Technol..

[12] Teleszko M., Wojdyło A., Rudzińska W., Oszmiański J., Golis T. (2015). Analysis Of Lipophilic And Hydrophilic Bioactive Compounds Content In Sea Buckthorn (*Hippophhaë rhamnoides* L.) Berries. J. Agric. Food Chem..

[13] Sarion C., Codină G.G., Dabija A. (2021). Acrylamide on Bakery Products: A Review on Health Risks, Legal Regulations and Strategies to Reduce its Formation. Int. J. Environ. Res. Public Health.

[14] Sung W.C., Chen C.Y. (2017). Influence of Cookies Formulation on the Formation of Acrylamide. J. Food Nutr. Res..

[15] Mesías M., Morales F.J., Delgado-Andrade C. (2019). Acrylamide in biscuits commercialised in Spain: A view of the Spanish market from 2007 to 2019. Food Funct..

[16] Mousavi Khaneghah A., Fakhri Y., Nematollahi A., Seilani F., Vasseghian Y. (2020). The Concentration of Acrylamide in Different Food Products: A Global Systematic Review, Meta-Analysis, and Meta-Regression. Food Rev. Int..

[17] Borczak B., Sikora M., Kapusta-Duch J., Fołota M., Szewczyk A., Zięć G., Doskočil I., Leszczyńska T. (2022). Antioxidative Properties and Acrylamide Content of Functional Wheat-Flour Cookies Enriched with Wild-Grown Fruits. Molecules.

[18] Ou J., Wang M., Zheng J., Ou S. (2019). Positive and negative effects of polyphenol incorporation in baked foods. Food Chem..

[19] Janotková L., Potočňáková M., Kreps F., Kepsová Z., Ácsová A., Ház A., Jablonský M. (2001). Effect of sea buckthorn biomass on oxidation stability and sensory attractiveness of cereal biscuits. BioResources.

[20] Kuchtová V., Kohajdová Z., Karovićová J., Lauková M. (2018). Physical, Textural and Sensory Properties of Cookies Incorporated with Grape Skin and Seed Preparations. Pol. J. Food Nutr. Sci..

[21] Assis L., Zavareze E.D.R., Radünz A.L., Dias Á., Gutkoski L.C., Elias M.C. (2009). Propriedades nutricionais, tecnológicas e sensoriais de biscoitos com substituição de farinha de trigo por farinha de aveia ou farinha de arroz parboilizado. Alim. Nutr. Araraquara.

[22] Hadiyanto, Asselman A., van Straten G., Boom R.M., Esveld D.C., van Boxtel A.J.B. (2007). Quality prediction of bakery products in the initial phase of process design. Innov. Food Sci. Emerg. Technol..

[23] Zbikowska A., Kowalska M., Zbikowska K., Onacik-Gür S., Łempicka U., Turek P. (2022). Study on the Incorporation of Oat and Yeast-Glucan Into Shortbread Biscuits as a Basis for Designing Healthier and High Quality Food Products. Molecules.

[24] Pathare P.B., Opara U.L., Al-Julanda Al-Said F. (2013). Colour Measuremet and Analysis in Fresh and Processed Foods: A Review. Food Bioproc. Technol..

[25] Criste A., Urcan A.C., Bunea A., Pripon Furtuna F.R., Olah N.K., Madden R.H., Corcionivoschi N. (2020). Phytochemical Composition and Biological Activity of Berries and Leaves from Four Romanian Sea Buckthorn (*Hippophae Rhamnoides* L.) Varieties. Molecules.

[26] Geng Z., Zhu L., Wang J., Yu X., Li M., Yang W., Hu B., Zhang Q., Yang X. (2023). Drying sea buckthorn berries (*Hippophae rhamnoides* L.): Efects of diferent drying methods on drying kinetics, physicochemical properties, and microstructure. Front. Nutr..

[27] Park M.G., Joo S.Y. (2021). Quality Characteristics and Antioxidant Activity of Rye Cookies Supplemented with Sea Buckthorn Leaf Powder. J. Korean. Soc. Food Sci. Nutr..

[28] Danielski R., Shahidi F. (2024). Phenolic composition and bioactivities of sea buckthorn (*Hippophae rhamnoides* L.) fruit and seeds: An unconventional source of natural antioxidants in North America. J. Sci. Food Agric..

[29] Karafyllaki D., Narwojsz A., Kurp L., Sawicki T. (2023). Effects of different processing methods on the polyphenolic compounds profile and the antioxidant and anti-glycaemic properties of horseradish roots (*Armoracia rusticana*). Eur. Food Res. Technol..

[30] Cheynier V. (2012). Phenolic compounds: From plants to foods. Phytochem. Rev..

[31] Sik B., Székelyhidi R., Lakatos E., Kapcsándi V., Ajtony Z. (2022). Analytical procedures for determination of phenolics active herbal ingredients in fortified functional foods: An overview. Eur. Food Res. Technol..

[32] Jabłońska M., Karpińska-Tymoszczyk M., Surma M., Narwojsz A., Reszka M., Błaszczak W., Sawicki T. (2024). Enrichment of shortcrust pastry cookies with bee products: Polyphe-nol profile, in vitro bioactive potential, heat-induced compounds content, colour parameters and sensory changes. Sci. Rep..

[33] Valdés A., Álvarez-Rivera G., Socas-Rodríguez G., Herrero M., Ibáñez E., Cifuentes A. (2022). Foodomics: Analytical opportunities and challenges. Anal. Chem..

[34] Chen L., Xin X., Yuan Q., Su D., Liu W. (2014). Phytochemical properties and antioxidant capacities of various colored berries. J. Sci. Food Agric..

[35] Dienaitė L., Pukalskas A., Pukalskienė M., Pereira C.V., Matias A.A., Venskutonis P.R. (2020). Phytochemical composition, antioxidant and antiproliferative activities of defatted sea buckthorn (*Hippophaë rhamnoides L*.) berry pomace fractions consecutively recovered by pressurized ethanol and water. Antioxidants.

[36] Altissimi M.S., Roila R., Branciari R., Miraglia D., Ranucci D., Framboas M., Haouet N. (2017). Contribution of street food on dietary acrylamide exposure by youth aged nineteen to thirty in Perugia, Italy. Ital. J. Food Saf..

[37] EFSA (2015). Scientific Opinion on Acrylamide in food. EFSA J..

[38] Commission Regulation (EU) No 2017/2158 of 20 November 2017 Establishing Mitigation Measures and Benchmark Levels for the Reduction of the Presence of Acrylamide in Food (Text with EEA Relevance). https://op.europa.eu/en/publication-detail/-/publication/1f3b45fb-ce6b-11e7-a5d5-01aa75ed71a1/language-en.

[39] International Agency for Research on Cancer (IARC) (1994). Some Industrial Chemicals.

[40] Zhang Y., Zhang G., Zhang Y. (2005). Occurrence and analytical methods of acrylamide in heat-treated food. Review and recent development. J. Chromatogr. A.

[41] WHO (2002). Health Implication at Acrylamide in Food.

[42] Swedish National Food Administration (2002). Information about Acrylamide in Food. http://www.slv.se.

[43] Kazanci M., Guner K.G., Durakli Velioglu S. (2024). The effect of the use of pekmez and honey as sugar substitutes on the quality characteristics and the acrylamide content of sponge cakes and cookies. J. Food Meas. Charact..

[44] Kukurová K., Rerková L., Belovic M., Perović L., Torbica A., Ciesarová Z. (2023). The impact of asparaginase on textural properties of wholegrain cereal biscuits enriched with sea buckthorn pomace. J. Microbiol. Biotech. Food Sci..

[45] Ghosh S., Falyouna O., Malloum A., Othmani A., Bornman C., Bedair H., Onyeaka H., Al-Sharify Z.T., Ajala Jacob O., Miri T. (2022). A general review on the use of advance oxidation and adsorption processes for the removal of furfural from industrial effluents. Microporous Mesoporous Mater..

[46] Sturza R., Ghendov-Moşanu A., Deseatnicov O., Suhodol M.N. (2016). Use of sea buckthorn fruits in the pastry manufacturing. Chem. Chem. Eng. Biotechnol. Food Ind..

[47] Horszwald A., Andlauer W. (2011). Characterization of bioactive compounds in berry juice by traditional photometric and modern microplate methods. J. Berry Res..

[48] Sawicki T., Błaszczak W., Latocha P. (2023). In vitro anticholinergic and antiglycaemic properties of frost-hardy Actinidia fruit extracts and their polyphenol profile, L-ascorbic acid content and antioxidant capacity. Food Res. Int..

[49] Sawicki T., Surma M., Sadowska-Rociek A. (2023). Characteristics of contaminants in the polish-origin bee products and cancer risk assessment. Food Chem. Toxicol..

[50] Chociej P., Foss K., Jabłońska M., Ustarbowska M., Sawicki T. (2024). The profile and content of polyphenolic compounds and antioxidant and anti-glycation properties of root extracts of selected medicinal herbs. Plant Foods Hum. Nutr..

[51] Gumul D., Ziobro R., Korus J., Surma M. (2023). Pulp from colored potatoes (*Solanum tuberosum* L.) as an ingredient enriching dessert cookies. Foods.

[52] (2003). Sensory Analysis—Guidelines for the Use of Quantitative Response Scales.

[53] (2023). Sensory Analysis—General Guidelines for Selection, Training and Monitoring of Selected Assessors and Expert Sensory Assessors.

[54] (2007). Sensory Analysis—General Guidance for Design of Test Rooms.

